# Small extrachromosomal circular DNA (eccDNA): major functions in evolution and cancer

**DOI:** 10.1186/s12943-021-01413-8

**Published:** 2021-09-03

**Authors:** Xiaoxuan Ling, Yali Han, Jinxue Meng, Bohuan Zhong, Jialong Chen, He Zhang, Jiheng Qin, Jing Pang, Linhua Liu

**Affiliations:** 1 Dongguan Key Laboratory of Environmental Medicine, School of Public Health, Guangdong Medical University, Dongguan, 523808 P.R. China; 2 Department of Preventive Medicine, School of Public Health, Guangdong Medical University, Dongguan, 523808 P.R. China

**Keywords:** Extrachromosomal circular DNA, Evolution, Cancer, Function of eccDNA

## Abstract

Extrachromosomal circular DNA (eccDNA) refers to a type of circular DNA that originate from but are likely independent of chromosomes. Due to technological advancements, eccDNAs have recently emerged as multifunctional molecules with numerous characteristics. The unique topological structure and genetic characteristics of eccDNAs shed new light on the monitoring, early diagnosis, treatment, and prediction of cancer. EccDNAs are commonly observed in both normal and cancer cells and function via different mechanisms in the stress response to exogenous and endogenous stimuli, aging, and carcinogenesis and in drug resistance during cancer treatment. The structural diversity of eccDNAs contributes to the function and numerical diversity of eccDNAs and thereby endows eccDNAs with powerful roles in evolution and in cancer initiation and progression by driving genetic plasticity and heterogeneity from extrachromosomal sites, which has been an ignored function in evolution in recent decades. EccDNAs show great potential in cancer, and we summarize the features, biogenesis, evaluated functions, functional mechanisms, related methods, and clinical utility of eccDNAs with a focus on their role in evolution and cancer.

## Introduction

Extrachromosomal circular DNA (eccDNA) refers to circular DNA that originate from chromosomes, but once generated, these DNAs are likely independent of chromosomal DNA. EccDNA residing in nuclei was first discovered by Alix Bassel and Yasuo Hoota in 1964 [1] and was referred to as double minutes (DMs). EccDNA was first identified by karyotyping as DNA that exists independent of the chromosomes in cancer cells [2] but has emerged as a new type of molecule because of their numerous characteristics and functions identified due to technological advancements, particularly next-generation sequencing and bioinformatics [3–5]. EccDNAs are sufficiently long to harbor their own origins of replication, are able to encode amino acids, and have been observed in tumor tissues, where they most frequently harbor oncogenes or genes related to drug resistance in cancer therapy [6–9]. Other functions of eccDNAs, such as functions in aging [10–12] and heterogeneity [13], as well as putative roles, including dosage compensation, have gradually been discovered. The genotype diversity and genomic plasticity within cancer cells are known to be affected by genome instability and genome alterations [14]. EccDNA can rapidly remodel the genome through its diversity, including structural, functional, and numerical diversity, which efficiently drives the evolution of cancer and life [6, 8, 9]. In this review, we discuss the characteristics, biogenesis, methodology, and mechanisms of eccDNA functions with a focus on their role in evolution and cancer.

## Molecular structure of eccDNA

### Size of eccDNA

The size of eccDNAs varies widely, ranging from dozens of bases to hundreds of thousands of bases; in addition, the majority are smaller than 1,000 bp [15, 16], and 99% of eccDNAs are shorter than 25 kb. More than 50% of eccDNAs originate from genic or pseudogenic regions [16]. Most of the eccDNAs found in normal cells are shorter and usually less than 500 bp [17–20]. However, the genetic content of eccDNAs shows marked differences among leukocytes from the same individual [16]. Fetal-derived eccDNAs are shorter than maternal eccDNAs [21], and fetal eccDNAs in plasma are relatively hypomethylated compared with maternal eccDNAs [22]. EccDNAs from tumors have smaller sizes and prefer end coordinates from those derived from normal cells [23–25]. In summary, the sizes and levels of eccDNAs exhibit lifestyle-, life-stage-, tissue-, and disease-specific features, and these findings shed new light on tracing the origin of eccDNAs [16, 26–28] and advancing cancer diagnosis and treatment methods.

### Distribution of eccDNA

**Fig. 1:**
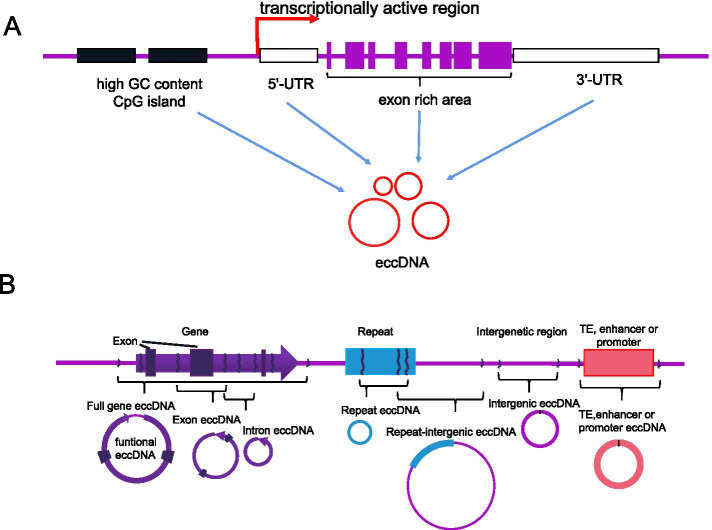
Origins and types of eccDNA. **A** EccDNA is enriched in specific areas (hotspots), including untranslated (3’-UTR and 5’-UTR) areas, regions with a high GC content, and transcriptionally active chromatin. **B** EccDNA types based on the genetic content [34]

The vast majority of eccDNA originates from repetitive sequences [17, 29–32]. EccDNAs with a length less than 500 bp are called small polydisperse circular DNA (spcDNA) [17, 29–32], and a few spcDNA molecules are hybridized to unique sequences [29, 31–33]. The eccDNA distribution is not restricted to particular areas in the genome [34]. EccDNAs originate from tens of thousands of unique sites in the genome and are enriched in specific areas (called hotspots), including untranslated areas and regions with a high GC content; however, in transcriptionally activated chromatin, eccDNAs prefer to reside in circularization hotspots, but this finding appears to be controversial (Fig. 1A) [15].

Gene-rich chromosomes contribute to more eccDNAs per megabase, e.g., gene-rich chromosomes 17 and 19 contribute to a higher average frequency of eccDNAs per megabase than other chromosomes; moreover, the ratio of eccDNAs per megabase to coding genes is positively correlated [16]. For example, the most transcribed protein-coding gene in muscle, titin (TTN), has the greatest amount of eccDNAs [16]. Noncoding gene areas show a lower correlation with the eccDNA frequency, which suggests that the transcription or other features of coding genes affect the frequency of eccDNA formation. A substantial portion of eccDNA sequences originate from helitrons (a class of mobile elements known to transpose via a circular intermediate) [35], cut-and-paste transposons, and exons.

### Genetic features and category of eccDNA

Genomic DNA flanking 80% of circular microDNA has direct repeats of 2–15 bases [15]. Approximately 60% of eccDNAs arise from unique sequences, and > 90% of genomic sites harbor direct repeats of several bases. The junction of these repeats creates a unique paired-end sequence, which is useful for the recognition of eccDNA through a bioinformatic analysis of sequence data [36]. The distribution of the eccDNA sequences in chromosomes is distant from each other in prostate and ovarian cancer cell lines, which indicates that the sites of eccDNA formation depend on the cell lineage [37]. EccDNAs have been categorized into the following 4 types according to their size and sequence: spcDNA, telomeric circle, microDNA, and eccDNA [3]. Based on their genomic origin and genetic content, we propose that eccDNAs can also be categorized into the following 7 types: full gene eccDNA, exon eccDNA, intron eccDNA, repeat eccDNA, repeat-intergenic eccDNA, intergenic eccDNA, transposable element (TE) eccDNA, and promoter/enhancer eccDNA (Fig. 1B). TE eccDNA and promoter/enhancer eccDNA can be reinserted into other types of eccDNAs to generate larger eccDNAs called function-enhanced eccDNAs because eccDNAs are able to gradually become enlarged through assembly from smaller circular elements [38–40]. These factors form the structural diversity of eccDNAs and serve as the genetic basis of their functional and numerical diversity.

## Biogenesis and replication of eccDNA

### Outline

EccDNAs may arise via many different pathways with different mechanisms. Some models of eccDNA formation, including the breakage-fusion-bridge (BFB) cycle, chromothripsis, episome model, translocation-deletion-amplification model, and ‘lost-and-found’ event of DNA [3, 21, 36, 37], have been proposed, and DNA breakage, DNA recombination, and DNA rearrangement play critical roles in these models [36, 41–43]. These models of eccDNA formation work collectively, but the details remain elusive. DNA replication, DNA transcription processes, and other events are also regarded as potential mechanisms [36]. Overall, eccDNA is generated from various processes, but further research related to DNA metabolism is needed.

### Breakage-fusion-bridge (BFB) cycle

Most eccDNA molecules include or are adjacent to short direct repeats, and 72.4% (human) and 8.7% (pigeon) of 30,000 unique eccDNAs, as characterized by eccDNA sequencing, are derived from repetitive elements [34]. In addition, specialized eccDNAs have also been noted to arise from repetitive genomic sequences, such as telomeric DNA or rDNA [36]. Furthermore, several researchers have found that some nonrepetitive spcDNA sequences are flanked on both ends by, on average, 9–11 bp of direct repeats [17, 31, 32, 44]. These findings suggest that DNA repair pathways, such as homologous recombination and microhomology-mediated end joining between two short repeats, may generate DNA circles.

**Fig. 2:**
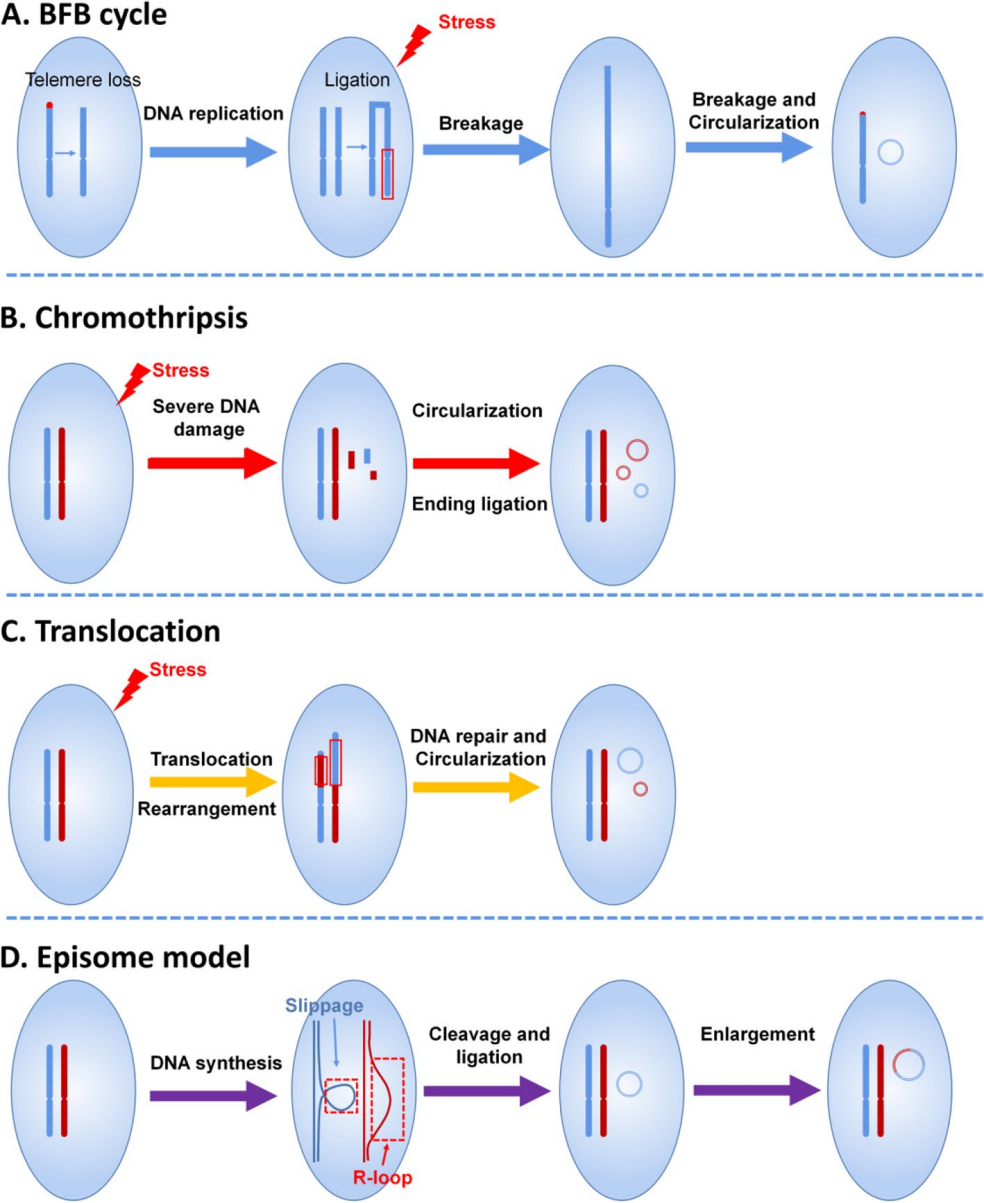
Mechanisms of eccDNA biogenesis. **A** A dicentric anaphase bridge forms due to the loss of telomeres, and the telomere-free bridge is elongated by a repetitive cycle of replication, broken into random fragments under stress, and looped out to form eccDNA. **B** Chromothripsis, a type of severe DNA damage caused by exogenous stress, forms single- or double-strand breakages. With the DNA repair system, most of the DNA fragments are removed by different mechanisms, but some of the fragments are ligated and circularized into eccDNAs. **C** The translocation-deletion-amplification mechanism, which is frequently triggered by exogenous stimuli, is repaired or removed by the DNA repair system, but the retained or cleaved DNA fragments may generate eccDNAs. **D** Circular DNAs are generated during the DNA synthesis process in a DNA slippage and R-loop manner. Circular DNAs are produced by cleavage and ligation and can be enlarged by the integration of other DNA components, such as TEs and enhancers/promoters. BFB, breakage-fusion-bridge

Repetitive DNA sequences can be excised by homologous recombination to generate larger eccDNAs [45, 46]. The formation of eccDNAs smaller than < 2 kb is considered a consequence of intramolecular recombination or rearrangement between adjacent repeats (telomeric, centromeric, and satellites) mediated by a double-strand break [15] and enlarged through the BFB cycle [3]. The mechanism of the BFB cycle is illustrated in Fig. 2A.

### Chromothripsis

Chromothripsis results from severe DNA damage induced by exogenous stimuli. The circularization of DNA fragments from chromosome breakage generates eccDNAs (Fig. 2B). EccDNA is derived from both coding and noncoding genomic regions and is present in normal physiological states [47]. However, some researchers have speculated that chromothripsis is one of the mechanisms of eccDNA formation. The chemotherapeutic-induced apoptosis of lymphoblastoid cells significantly promotes eccDNA production [41], but eccDNAs are not byproducts of apoptosis-driven fragmentation [16]. It is well known that most carcinogens are mutagens that lead to DNA damage. The accumulation of eccDNA has been found after exposure to the carcinogen 7,1-dimethylbenz[a]anthracene and the DNA replication inhibitor hydroxyurea [17]. Cycloheximide, an inhibitor of protein synthesis, also induces 70-fold elevation of eccDNAs in murine cells [17]. Human cells with a specific defect in the DNA repair pathway produce an increased number of eccDNA molecules [30]. The deletion of MSH3, a gene involved in the DNA mismatch repair pathway, decreases the eccDNA levels to 80% [37]. The CTC1/STN1/TEN1 protein complex, which is known to participate in the maintenance of telomeres, has been shown to contribute to t-circle formation [48]. Prokaryotic cells with SGS1, a protein involved in DNA repair, have increased eccDNA levels [49]. EccDNA can be generated by CRISPR/CAS9 technology, which supports the notion that the DNA repair pathway participates in the formation of eccDNAs [43]. These findings suggest that DNA damage is involved in the biogenesis of eccDNAs, but the underlying pathways and mechanisms need further investigation.

### Translocation-deletion-amplification mechanism

The translocation-deletion-amplification mechanism, which is frequently triggered by exogenous stimuli, may be repaired or removed by the DNA repair system. The retained or cleaved DNA fragments may generate eccDNAs (Fig. 2C). Gene amplification adjacent to a breakage point is more frequent, as supported by the amplification of MYC-containing eccDNA [50].

### Episome model

A research group recently showed that MYC-containing DMs in leukemia cases arise by excision and amplification, and this finding supports the eccDNA formation model called the episome model [38] (Fig. 2D). This amplification is mediated by small circular DNAs, which are referred to as episomes. An episome is likely an unrecognized type of structural variation in the genome, but findings from other research studies have noted a possible role of circular DNAs in the movement of TEs [51]. The frequency of these elements in cancer cells is higher than that in normal cells, which indicates that these elements might contribute to the instability of the genome. The consistency between eccDNAs generated through recombination events within the genome and their recombination junctions supports the possibility that retrotransposable elements may move around the genome through DNA circularization [52]. EccDNAs from DNA circularization result in gene copy number variations when circles contain genes and origins of replication [53], which contributes to additional gain of novel functions. For example, a previous study revealed the eccDNA-based amplification and transmission of herbicide resistance in the crop weed Amaranthus palmeri [54]. Most retrotransposable elements move through a copy-and-paste or cut-and-paste mechanism, and this movement is likely to have a functional impact on the genomic context [51]. For example, transposon-like sequences, called telomere-bearing elements (TBEs), in Oxytricha are excised as eccDNA molecules during rearrangement, which results from rejoining to regenerate the target [55] (Fig. 2D). The accumulation of eccDNAs in normal cells from GC-rich and transcriptionally activated regions of the genome suggests that R-loop generation and repair may be involved in the formation of eccDNA [37].

### Role of DNA replication in eccDNA biogenesis

A review has proposed that replication slippage during DNA replication is also involved in the formation of eccDNA [56]. Replication slippage is commonly mediated by direct repeats in the genome but cleaved by the DNA repair system, which ultimately generates DNA circles. Because this notion is partially supported by little evidence, further investigation is needed.

Collectively, these studies suggest that the formation of eccDNA is dependent on the sequence, organization, replication, and damage repair of DNA. However, some researchers argue that the formation of eccDNAs may occur in the absence of any repetitive DNA elements, probably using a microhomology sequence of 8 nucleotides flanking the amplified sequence [36].

### Role of eccDNA replication in eccDNA biogenesis

Whether eccDNA replication is independent of cell proliferation remains elusive. EccDNAs appear to undergo extrachromosomal replication via a rolling circle mechanism [56]. An estimate, probably an underestimate, of the eccDNA number has been quantified by electron micrographs (EMs) using preparations from defined numbers of cells, and the results suggest the existence of at least 125 ~ 200 DNA circles per DT40 cell [37]. The contribution of DNA replication to eccDNA production, however, is controversial; some studies have found that the eccDNA levels increase when ongoing replication is blocked by replication inhibitors [17], whereas other studies have found that eccDNA can be formed in the absence of any DNA replication [45]. However, we speculate that replication is independent of mitosis, which helps cells survive under challenging conditions, such as hazardous chemical exposure or nutrient deficiency, and is thus not suitable for mitosis.

## Function and mechanism of eccDNA

**Fig. 3:**
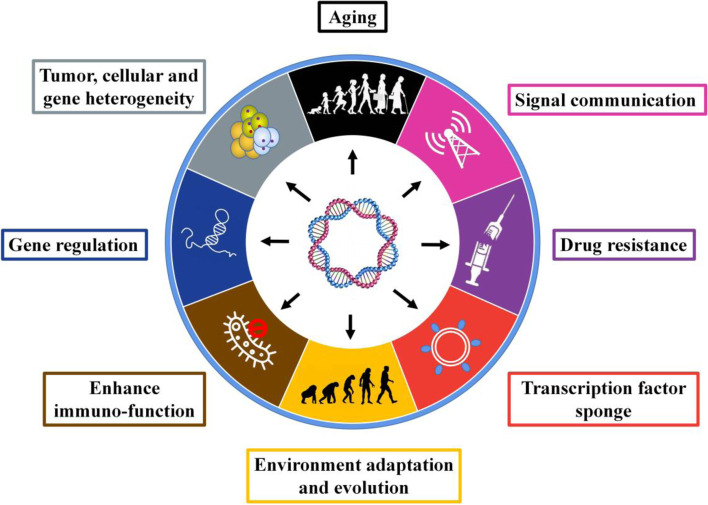
Identified functions of eccDNA. Increasing studies have implied the great promise of eccDNA in cancer research because the identified functions work synergistically in cancer initiation and progression

EccDNAs play important roles in genetic variation, evolution, genomic instability, genomic plasticity, drug resistance, environmental adaption, mutation, and tumorigenesis. Some identified functions of eccDNA have been summarized in a previous review [36]. We updated and reorganized the recently identified roles of eccDNA (Fig. 3). Genetic variation is the fundamental change of other aspects. Gene amplification is a type of genetic variation whose role has been validated in various biological processes, and the reintegration of eccDNAs provides an efficient pathway for gene amplification [36, 43]. EccDNA may contribute to variations in the genome content through evolution [44]. In both human tumor cell lines and yeast, the significant correlation between the appearance of eccDNAs and the selective growth and/or resistance advantage of cells indicates that eccDNAs influence the phenotype of cells. In yeast, eccDNAs participate in gene amplification [57] and thereby facilitate adaptation to nutrient-limiting environments [42, 58, 59], specific amino acid limitation [60], aging [10–12], and senescence caused by rDNA circles [61]. Similarly, in plants, eccDNA plays a role in transmissible herbicide resistance [54], and 49 of the 59 genes encoded by the eccDNA replicon are transcriptionally active when replicon-containing glyphosate-resistant plants are exposed to glyphosate [62]. In human tumor cells, eccDNA may drive increases in the oncogene copy number to yield a high level of oncogene products [38, 63]. Some researchers have speculated that eccDNA may contribute to the expression of different isoforms of a gene by interfering with or promoting the transcription of specific exons [15]. The theoretical functions of eccDNA include the expression of regulatory RNAs that sponge transcription factors, as has been partially validated [64], and this finding suggests that one of the underlying mechanisms through which eccDNA drives gene expression involves genetic variation, such as gene amplification and rearrangement. Moreover, eccDNA-containing oncogenes show significantly higher amplification through the mechanism of eccDNA formation than through chromosomal amplification [65], but this mechanism is not the only function of eccDNA in its biological roles. A recent study found that small artificial eccDNAs suppress gene expression by producing short regulatory RNAs [64]. Collectively, the published studies show that the role and mechanism of eccDNAs are dependent on the gene content they contain and the structures of the elements. EccDNA provides genetic plasticity and heterogeneity driven by extra chromosomes, which remains an ignored power of evolution in recent decades.

**Fig. 4:**
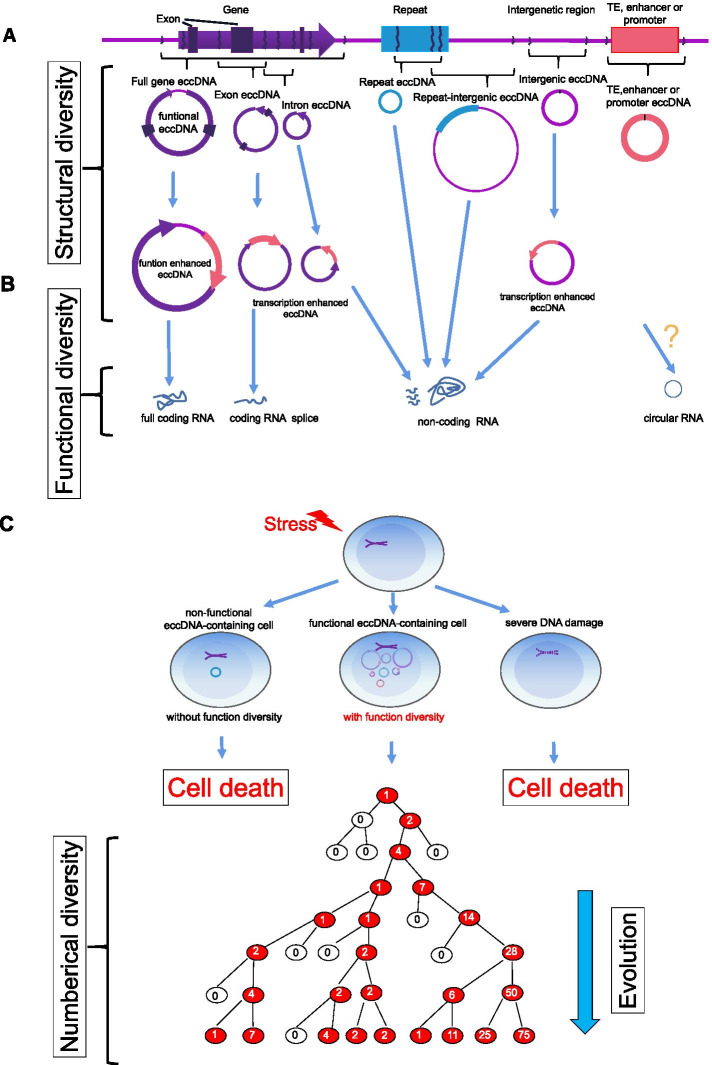
Diversity of eccDNA and its roles in evolution. **A** Structural diversity of eccDNA. Different types of eccDNA have different genetic contents, which constitute the eccDNA structural diversity. **B** Functional diversity of eccDNA. The eccDNA structural diversity and unique topological structure contribute to the multiple functions of eccDNA by potentially driving the expression of coding RNAs, noncoding RNAs, and other RNAs. **C** Numerical diversity of eccDNA as well as the role and mechanism of eccDNA in evolution (modified based on Verhaak et al. [71]). Cells with eccDNA functional diversity formed from different pathways triggered by exogenous stimuli have an enhanced opportunity to generate eccDNA numerical diversity, which is attributed to cell survival advantages in environmental adaptation and evolution. The cycle number represents the copy number of eccDNA

Based on the defined characteristics of the genetic structure of eccDNA, we propose that the diversity of eccDNAs, including the structure, number, and function of diversity, is an important origin driving the evolution of the genome and life (Fig. 4). To ensure a better understanding, we consider all the eccDNAs in cells or organisms to be adaptive toolkits that may respond to any possible stress caused by the environment. Each eccDNA plays a unique role in coping with the stimulus immediately or after its evolution because loosened chromatin facilitates eccDNA-encoding gene products more efficiently. Exposure to environmental stimuli results in the generation of various eccDNAs that form the original diversity of eccDNA. Although most eccDNAs initially have no adaptive advantages, eccDNAs eventually have the chance to obtain the genomic architecture and are prone to gain adaptive advantages to facilitate the evolvability of cells exposed to varied levels of environmental stimuli or nutrients, which results in the formation of countless eccDNAs from many alternations in DNA [60]. Once functional eccDNA is generated in a somatic cell, the structural diversity and amount are maintained even though many nonfunctional eccDNAs are present in cells. Moreover, eccDNAs generally separate randomly during mitosis due to the absence of a centromere [8] and replicate independent of cell mitosis [66], which causes significant heterogeneity in complement eccDNAs across the population and thereby results in substantial phenotypic plasticity [8]. Gene copy number amplification is increased much faster via the accumulation of eccDNA rather than chromosomal DNA [65]. Population heterogeneity can be selected according to the dosage of eccDNAs, not only by an increased dosage [8, 67] but also by a decreased dosage, due to the ease of eccDNA loss during cell division [68–70].

EccDNAs derived from gene-rich chromosome regions may influence the genotype of somatic cells through the alteration of gene copy numbers and the transcription of full-length genes, truncated genes, or regulatory RNAs (Fig. 4B) [16]. Moreover, the eccDNA diversity identified in a previous study supports a model in which any part of the human genome can contribute to eccDNA [16]. These findings have also been validated by the previous finding that the oncogene MET in glioblastoma cells is amplified on eccDNA molecules, as observed by fluorescent in situ hybridization (FISH) [8].

EccDNAs are transcriptionally activated and contribute to phenotypic variation by driving full-length and/or truncated gene expression, and during this course, regulatory RNAs may also be transcribed [16]. The underlying mechanism may include loosened chromatin, variations in the topological DNA structure, epigenetic changes, and the reintegration of promoters, enhancers, and TEs. EccDNAs can be categorized into several types, including full-gene eccDNA, exon eccDNA, intron eccDNA, repeat eccDNA, and intergenic eccDNA, depending on their sequence contents, which can be transcribed into all defined transcripts, including mRNA, microRNA, long noncoding RNA, and circular RNA.

In summary, the theoretical and defined functions of eccDNA as well as their mechanisms include aging, signaling communication, drug resistance, sponging of transcription factors, environmental adaption via adaptive DNAs, stimulation of immunofunction, gene regulation by expressing regulatory RNAs, contribution to heterogeneity through diversity, and gene dosage compensation [57]. More functions should be revealed via further investigations (Fig. 4), and the evaluated functions related to cancer and drugs are summarized in Table 1.

**Table 1:** Summary of functions of eccDNAs related to cancer

Type of cancer	Genes	Drugs/Treatment	Biogenesis	Function	References
Pancancer (17 different cancer types)	EGFR	Erlotinib	Rearrangement	Contributes to intratumoral heterogeneity via the reintegration of EGFRvIII-containing eccDNA elements and by promoting the transcription of EGFR; additional rearrangements and heterogeneity after erlotinib withdrawal.	[65]
Pancancer (17 different cancer types)	MYC	NA	Rearrangement	Contributes to intratumoral heterogeneity by promoting the transcription of MYC.	[65]
Pancancer (prostate cancer, colon cancer, glioblastoma)	EGFR, MYC, CDK4 and MDM2	NA	NA	Promotes the expression of oncogenes (EGFR, MYC, CDK4, and MDM2) encoded on eccDNA by influencing the chromatin organization.	[7]
Glioblastoma	EGFR	Irradiation	NA	Cells with amplified EGFR on eccDNA exhibit stronger invasive properties and radiation resistance.	[72]
Glioblastoma	MET	Capmatinib	NA	Amplified MET on eccDNA drives early tumor formation, and the elimination of eccDNA increases the survival benefit.	[8]
Glioblastoma	MYC	NA	NA	EccDNA harboring MYC amplification contributes to recurrent tumors.	[8]
Glioblastoma	EGFR	Dacomitinib	NA	EccDNA harboring EGFRvIII mutation amplification drives recurrent tumors upon treatment with dacomitinib.	[8]
Glioblastoma	EGFRvIII	Temozolomide with adjuvant radiation	NA	EccDNA containing EGFRvIII provides cells with growth advantages.	[73]
Glioblastoma	MDM2	Erlotinib	NA	The amplification of MDM2 on eccDNA promotes erlotinib resistance.	[70]
Neuroblastoma	MYCN	NA	Involves neo-topologically associated domains	The hijacking of enhancers and insulators drives the expression of MYC on eccDNA.	[40]
Neuroblastoma	MYCN	NA	DNA repair or replication-associated mechanisms	Drives oncogenic genome remodeling and the expression of oncogenes.	[6]
Neuroblastoma	MYCN	Hydroxyurea	NA	The elimination of amplified MYCN on eccDNAs increases the sensitivity to hydroxyurea.	[74]
Cervical cancer	DHFR	Methotrexate	Chromothripsis, BFB	Adaptation to increased selection pressure is induced by methotrexate by increasing the DHFR gene copies in eccDNA, which promotes DHFR expression.	[43]
Cervical cancer	DHFR	Methotrexate	BFB	The amplification of DHFR located on eccDNA promotes resistance to methotrexate.	[75]
Breast Cancer	DHFR	Methotrexate	NA	Irradiation induces methotrexate resistance due to eccDNA with amplified DHFR.	[76]
Oral squamous cell carcinoma	MDR1	Hydroxyurea	NA	Loss of MDR1-carrying eccDNA induced by hydroxyurea increases drug sensitivity.	[77]
Colon cancer	DHFR	Methotrexate	NA	The elimination of DHFR-containing eccDNA promotes sensitivity to methotrexate and inhibits proliferation.	[78, 79]
Colorectal cancer	Not define	NA	Chromothripsis, a process of multistep evolution that drives eccDNA formation	eccDNA may drive cancer progression.	[80]
Leukemia	MYC	Hydroxyurea	NA	Hydroxyurea inhibits tumorigenicity by eliminating amplified MYC on eccDNAs.	[81]
Leukemia	c-Myc	Hydroxyurea and retinoic acid	NA	Hydroxyurea inhibits tumorigenicity by eliminating c-Myc-bearing eccDNAs.	[82]
Undefined	microRNA	NA	NA	Expresses functional small regulatory RNA.	[64]

*NA* Not available

## Methodology for eccDNA research

Previously, the de novo discovery of eccDNA was performed by EMs, Giemsa staining of metaphase chromosomes (karyotype), or two-dimensional (2D) gel electrophoresis, but these methods provide little information on eccDNA sequences. EccDNA was first detected and characterized by EMs with the generation of eccDNA-specific libraries [83]. The approximate size of eccDNAs was clearly identified by EMs, but data on the eccDNA number were rarely obtained [84]. Moreover, finding sequence information for eccDNAs is difficult [56]. Based on nucleotide pairing, other techniques, such as Southern blotting, inverse PCR, and FISH, provide evidence only about specific eccDNA elements. FISH, EMs, and Giemsa staining of metaphase chromosomes can provide localization information in cells. CsCl gradient purification and EM imaging approaches have been applied to investigate eccDNAs from several other organisms [15, 85–91]. Another research group verified the existence of DMs in human cancer cells by karyotype preparations and CsCl gradient purification [92, 93]. Fluorescence microscopy has been used to quantify changes in large eccDNAs and DMs between cancerous and normal cells [65]. An image analysis software package combined with fluorescence imaging has been used to quantify eccDNA copies [65]. Neutral–neutral 2D gel electrophoresis has been widely used for the identification and characterization of DNA replication forks [94, 95]. Several micrograms of genomic DNA is sufficient for these analyses, and pretreatment with restriction enzymes or exonucleases and the use of low-molecular-weight fractions of genomic DNA [96, 97] can improve the separation and resolution of eccDNA [98, 99]. In addition, 2D gel electrophoresis has been used for the measurement and characterization of eccDNAs [100] and has expanded the exploration of smaller eccDNAs [101]. However, most eccDNAs detected by 2D gels exist in the form of open circles. EccDNA detection using a 2D gel is insensitive, particularly for eccDNA with a supercoiled structure, and 2D gels are unable to discriminate dispersed repeats within a population of eccDNAs [56].

The sequence information of all types of known eccDNAs in a population of cells remains limited, and the technical limitations of the above-mentioned methods hinder advancements to clarifying aspects of eccDNAs [56]. Thus, a high-throughput sequencing technique with pretreatment and bioinformatics algorithms addressing these issues in eccDNA analysis has been recently developed. Pretreatment enhances the efficiency of the separation of linear and circular DNA [102, 103]. Bowtie 2 is a computational tool based on next-generation sequencing with bioinformatics algorithms for eccDNA identification and downstream analyses. Bowtie 2 (version 2.2.25) aligns the paired-end reads to the nematode (ce10) or human (hg38) reference genomes [104]. Picard deduplicates the mapped reads, and SAMtools sorts and indexes the unique reads. A separate positioning approach using both unique and repeated k-mer sequences is used to process the sequences that cannot be uniquely mapped [105]. The repetitive elements found in eccDNA fractions can be divided into unique chromosomal, focal, dispersed, and intrachromosomal repeats based on Dfam databases [106].

Advances in sequencing technologies have allowed the genome-scale identification and mapping of eccDNAs that range in size from 0.1 kb to 2 kb from human cell lines and mice [15, 37]. The commonly used cloning techniques with high resolution in analyzing eccDNAs are sufficiently sensitive under various conditions, which makes them less representative with respect to the sequence content and organization of eccDNA [56].

**Fig. 5:**
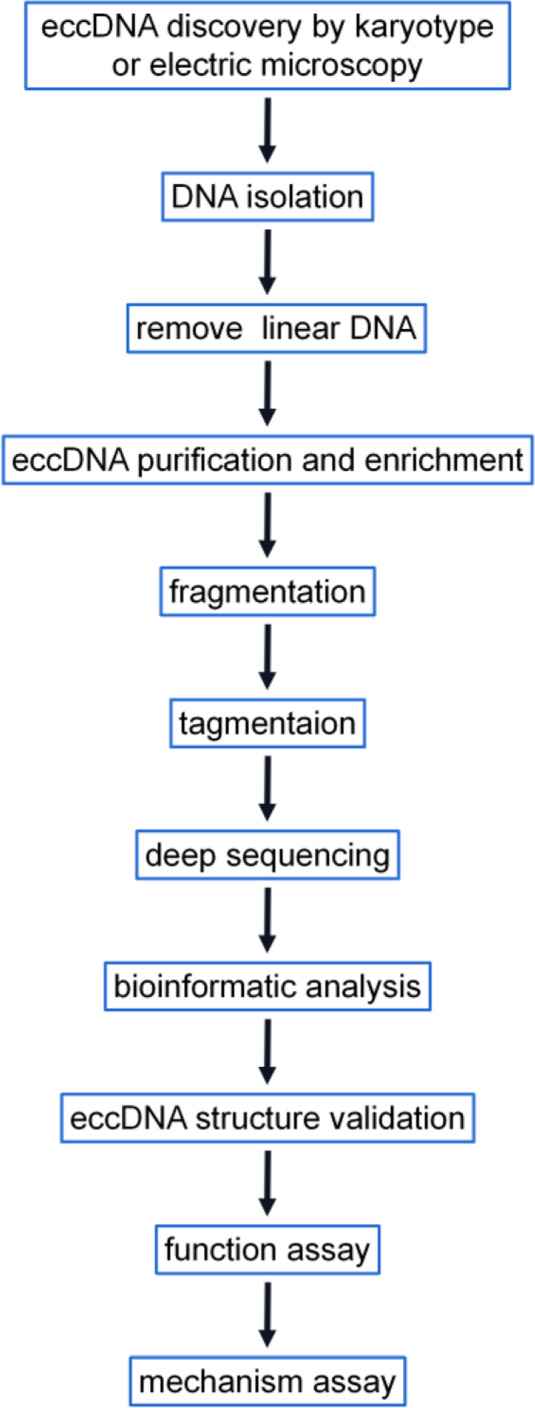
Flowchart for eccDNA research. The schematic comprehensively details eccDNA research, and some of the steps are not essential. In their research, researchers can select some of the steps based on the strengths and limitations of the methods listed in Table 1. Karyotyping and EMs, which are classic techniques in most labs, can qualitatively identify eccDNAs. Deep sequencing shows numerous strengths but is expensive. The process of eccDNA structure validation, which can be achieved by an integrated approach combining FISH and inverse PCR, is critical for studying eccDNA. Functional and mechanistic assays include transcriptomic, proteomic, and other approaches

Due to the low abundance of eccDNA, the purification of supercoiled circles on cesium chloride ethidium bromide (CsCl-EtBr) density gradients requires a large amount of biomaterials and has a low yield of open circles, which is the primary form of eccDNA [56]. A recent study used an approach based on CsCl-EtBr followed by tagmentation and high-throughput sequencing [47], and this approach overcomes the reduced yield of eccDNAs obtained by enrichment using exonuclease. Researchers have developed DNA topology-dependent approaches for enrichment and characterization, and this approach provides a comprehensive and robust profile of eccDNAs [47]. The high-throughput sequencing of exonuclease-resistant, rolling circle-amplified sequences followed by a paired-end computational method to identify junctional sequences allows the characterization of eccDNA sequences at base pair resolution [15]. Using these methods, however, the number of longer eccDNAs may be underestimated because smaller circles are amplified at a higher rate than larger circles even though EM suggests that most of the circles are small [37]. Digestion with Msp I requires a CCGG recognition site, which limits the proportion of eccDNAs that can be measured [21]. Furthermore, this approach might theoretically be biased toward larger eccDNA molecules due to a greater chance of containing such recognition sites. Tagmentation is more sensitive than the Msp I digestion method, particularly for shorter eccDNAs. On average, only 19.76% of the eccDNA molecules identified by the tagmentation approach have at least one CCGG motif. Thus, only approximately one-fifth of eccDNAs identified by the tagmentation approach are potentially detectable using the Msp I method [21]. Methods based on exonuclease III digestion can quantify covalently closed circular DNA [26]. We should note that eccDNAs are often unrecognized or lost by whole-genome studies relying on existing methods. The use of appropriate methods for eccDNA research is necessary for obtaining accurate results. Finally, we summarize the goals, advantages, and limitations of the methods that have been applied in eccDNA research (Table 2) and organize this information into a flowchart (Fig. 5).

**Table 2:** Overview of methods for eccDNA investigation

Methods	Description	Advantage or Limitation	References
Electron microscopy	Localization, identification, quantification	No sequence information	[9]
Karyotype	Localization, identification, quantification	No sequence information	[7]
2D gel electrophoresis	Identification	No sequence information	[98, 100]
Southern blotting	Quantification	Little sequence information	[98]
Inverse PCR	Quantification, identification	Little but useful sequence information	[15, 42]
FISH	Localization, identification, quantification	Little sequence information	[7–9]
Density gradient centrifugation	Isolation and purification	Laborious	[47]
Methods based on next-generation sequencing	Localization, quantification, high resolution, (1) tagmentation method is more sensitive than (2) nuclease digestion (Msp); (3) exonuclease III digestion; (4) exonuclease V digestion; (5) rolling-amplification	High throughput, high resolution, sensitive, expensive	[4–9, 26, 34]

## Perspective and challenges

EccDNAs contribute to several roles, including cell genotype, heterogeneity, and adaptation to environmental factors, such as lifestyle, nutrition, and stress. Therefore, the characteristics of tumor-specific eccDNAs in tumor and matched specimens may be helpful for the diagnosis and prognosis of these diseases. Recent studies have shown that the size distributions [107], end locations [108], end motifs [109], and epigenetic features [22] of eccDNAs are helpful for tracing their origin. EccDNAs, including those larger than 2 kb [110], can be released as extracellular free DNAs from healthy and unhealthy cells into biological fluid under different circumstances and may serve as novel biomarkers to shed new insights for the early detection of cancer, the monitoring of responses to drug treatment, and cancer survival [110–114]. We summarize the potential clinical utility of eccDNA in cancer in Table 3 based on differences in the copies, sequences, structure, and function of eccDNAs. However, the identification of specific eccDNAs with different functional roles requires additional investigation, which depends on novel tools and method innovation. More attention should be given to the functions and relative mechanisms of eccDNAs as well as the associated methodology because the discovery of and further research on eccDNAs are challenges of sequencing technology, which still cannot discriminate eccDNA from chromosomal DNA.

**Table 3:** Summary of the clinical utility of eccDNA

Clinical utility	Type of cancer	References
• Modulates the copies of oncogene-harboring eccDNAs to improve the efficacy of cancer treatment.		
(I) The elimination of eccDNAs carrying oncogenes promotes drug sensitivity.	Neuroblastoma	[74]
(II) Chemotherapeutic drugs promote the elimination of eccDNA containing amplified genes.	Neuroblastoma, colon cancer	[74, 115]
(III) DNA-PKs or PARP inhibitors decrease drug resistance and eccDNA production.	Colorectal cancer	[43]
• EccDNAs serve as promising biomarkers for cancer monitoring and prognosis.		
(I) Normal and cancerous tissues release eccDNAs into the circulation, which suggests promising potential for liquid biopsy.	Ovarian cancer, lung cancer	[110, 113]
(II) Patients with eccDNA carrying amplified MYCN exhibit worse overall survival than patients with MYCN-amplified tumors lacking such rearrangements.	Neuroblastoma	[6]
(III) EccDNA in the circulation is sharply decreased after surgery in patients with cancer.	Ovarian cancer, lung cancer	[110]
(IV) EccDNA is responsible for tumor recurrence.	Glioblastoma multiforme	[72]
• EccDNA serves as a promising tool for tracing the origin of the primary cancer.		
(I) The presence of eccDNA in the circulation is helpful for discriminating the origin of primary cancer.	NA	[114]
(II) The methylation status of eccDNA in the circulation is different and unique under specific physiological conditions, which makes eccDNA a trace biomarker.	Not applicable	[22]

*NA* Not available

## Data Availability

The datasets used and/or analyzed during the current study are available from the corresponding author upon reasonable request.

## Abbreviations

eccDNA: Extrachromosomal circular DNA
DMs: Double minutes
spcDNA: Small polydisperse circular DNA
cfDNA: Cell-free DNA
UTR: Untranslated region
BFB: Breakage-fusion bridge
TE: Transposable element
TBEs: Telomere-bearing elements
FISH: Fluorescent in situ hybridization
EMs: Electron micrographs
